# Menopause at work—An organisation‐based case study

**DOI:** 10.1002/nop2.2058

**Published:** 2023-12-14

**Authors:** Camille Cronin, Joanne Abbott, Nestor Asiamah, Susan Smyth

**Affiliations:** ^1^ School of Health & Social Care University of Essex Colchester UK; ^2^ North East London NHS Foundation Trust Rainham, Essex UK

**Keywords:** case study research, menopause, workplace

## Abstract

**Aim:**

The aim of the study was to explore and understand the organizational culture of a workplace in terms of support and well‐being for staff experiencing perimenopausal and menopausal symptoms at work.

**Design:**

It is widely acknowledged that perimenopause and menopause symptoms are experienced by a large percentage of the female workforce. There is a lack of research into how nurses are supported through menopause (Cronin et al. *Issues in Mental Health Nursing*, *42*, 2021, 541–548). The perimenopause and menopause transition can be a challenging time where many may require symptom management and support (RCN, The Menopause and Work: Guidance for RCN Representatives, 2020). This paper presents a case study research (CSR) approach to examine one healthcare organization.

**Methods:**

CSR design was used: A survey distributed to all staff employed, a review of the available documentation on menopause and interviews with managers from different levels of the organization. The COREQ consolidated criteria was used for reporting the qualitative research reported this study.

**Results:**

The case study generated both quantitative and qualitative data using surveys, interviews and documentation. Data from the organization (*n* = 6905) showed a majority female workforce of 81.9% with 40.6% aged between 41 and 55 years old, meaning a third of the organization working through perimenopause and menopause. Survey responses (*n* = 167) collected biographical and psychometric data on the prevalence of perimenopausal and menopausal symptoms. Seven managers were interviewed highlighting two themes: Access to support and culture of menopause and 13 documents from the organization on menopause were analysed for content. The study design permitted an iterative approach to data collection and providing an in‐depth understanding of the needs and support for those experiencing perimenopause and menopause. The findings help healthcare organizations to understand their workforce and take in to account the larger numbers of female employees particularly nurses with the need to provide person‐centred support mechanisms and an organizational approach for all employees.

## 1 INTRODUCTION

In the United Kingdom, menopause occurs around age 51 and there are more than 4.3 million working women aged 45–60, which means a huge number of women are working through the menopause transition (Hardy et al., 2019). While some women experience a problem‐free transition, around 80% of women will experience some menopausal symptoms, with a third experiencing severe symptoms such as hot flushes, insomnia and low mood (Hardy & Hunter, 2021). It is in the workplace where women find their symptoms most difficult to manage, embarrassment, stigma and fear that they might be discriminated against or stigmatised at work (Bazeley et al., 2022; Hardy et al., 2019).

This paper details a study of menopause in one organisation using case study research (CSR).

## 2 CASE STUDY RESEARCH

CSR is used to generate an in‐depth, multi‐faceted understanding of a complex issue in the real‐life context (Yin, 2017). It is an established research design that is used extensively in a wide variety of disciplines, particularly in the social sciences however controversy remains (Yazin, 2015), however Cronin (2014) argued it can be used as a rigorous form of inquiry. Today CSR continues to be a popular research method in many fields to investigate a wide range of phenomena and with advances in technology and methodology continue to make CSR a valuable tool for exploring complex issues in depth.

There are many influential authors and researchers who have contributed to the development of CSR. From an epistemology viewpoint, case study researchers must capture the knowledge and experience of the reality of the case and CSR provides a framework for this and the researcher must choose the right methods to investigate. CSR can be defined in a variety of ways but has one guiding principle; to explore an event or phenomenon in depth and in its natural context (Yin, 2017). Stake (1995) sees learning about the case and its processes as integral, whereas Merriam (1998) sees the study of the case with clear boundaries.

According to Yin (2017), case studies can be used to explain, describe, or explore events or phenomena in the everyday contexts in which they occur. This can help to understand and explain causal links around the case resulting in policy development or initiatives for service redesign. CSR captures information through exploration and understanding ‘how’, ‘what’ and ‘why’ things happen in the phenomena of that case under investigation (Yin, 2017). An important part of CSR is defining the case and formulating the research question(s) that are informed by existing literature or prior understanding of existing theory and settings.

The selected case should allow the research team access to the group of individuals, the organisation or the processes which will form the chosen unit of analysis for the study. A case could be pre‐selected for the researcher, with decisions being influenced by key stakeholders or because of empirical research leading to the rationale for choice and thereby needing access to a specific site. For example, this case was based on empirical evidence where Cronin et al. (2023) qualitative study explored the experiences of nurses and menopause in the workplaces across six countries. It aimed to explore perspectives on digital interventions for strategies to support menopausal women and found that while menopause is a transition for all women, there needs to be more recognition at work because it can impact work performance and consequently staff turnover. Cronin et al. (2023) research found participants reported the effects of menopausal symptoms and worried about workplace performance and patient care. The authors argued that improving women's well‐being and the ability to remain at work should be a priority and thus creating an open, supportive and inclusive culture for menopause. Therefore, it felt prudent to examine this further.

This paper therefore reports in a study where CSR was used to explore and understand the organisational culture of one UK healthcare organisation in terms of support and well‐being for staff experiencing perimenopausal and menopausal symptoms at work.

## 3 CONTEXT

Menopause typically occurs in the midlife period between the ages of 45 and 55 years (NHS England, 2022), with the average age of women reaching menopause in the United Kingdom by 51–52 year though women can experience menopausal symptoms outside this timeframe, with 1 in 100 women experiencing menopausal symptoms before the age of 40 (NHS England, 2022; NICE, 2022). Menopause is perceived to be a natural transition in a woman's life which includes three stages, perimenopause, menopause and post‐menopause; this transition can start up to 5 years prior to the last menstruation and is completed after 12 months without menstruation (Royal Collage of Nursing, 2020). Schaedel and Ryder (2022) report that while menopause is a natural part of ageing affecting 50% of the population, this fact is not reflected in society or our healthcare systems; and consequently, menopause is a neglected life event. Furthermore, Office for National Statistic (2021) reported the median age of death for UK women is 85.8 years, which will mean a significant majority of women will live their life in the postmenopausal phase. The lack of teaching on menopause in schools and minimal education for healthcare professionals has only exacerbated this issue contributing to the lack of knowledge and its negative impact on attitudes and perceptions in women's health (Aljumah et al., 2023).

The global population is ageing and living longer (World Health Organisation (WHO), 2007) meaning people are working and employed longer. UK working women aged over 45 form the fastest growing workforce and Office for National Statistic (2021) estimated by mid‐2019 there would be over 7 million women aged 45–60 working and working one third of their lives with menopause (Lobo & Gompel, 2022). Noticeably, women adopt more roles in the caring professions like nursing, teaching, and care work (Devine & Foley, 2020). In the United Kingdom, it is reported that 89.3% registered nurses are female and 10.7%; male, with this higher percentage of female working past the age of 45 years and likely to experience to impact of menopause in the workplace (Royal Collage of Nursing, 2020). Consequently, understanding the health related to the menopause transition should be a focus for all global women health strategies.

## 4 METHOD

The study set out to explore and understand the organisational culture of the workplace in terms of support and well‐being for staff experiencing perimenopausal and menopausal in one local organisation aiming to:

- To understand the prevalence and severity of perimenopausal and menopausal symptoms in employees.
- To explore factors that may be influencing these symptoms and their severity.
- To examine how the organisation might help employees whose working lives are impacted by perimenopausal and menopausal symptoms.

The goal of CSR is to create an accurate description of the case with data being collected prospectively with the phenomenon under investigation embedded in everyday practice, by taking a snapshot of time (March to June 2022). To ensure the CSR methods were appropriate for this organisation, a service user group established from the UK arm of the primary study (Cronin et al., 2023) had been formed and were already meeting on a regular basis. As a group they were approached to discuss, inform the design and test the tools. This proved useful to ensure language, user information and flow of the online tool was easy and accessible to use.

**FIGURE 1:**
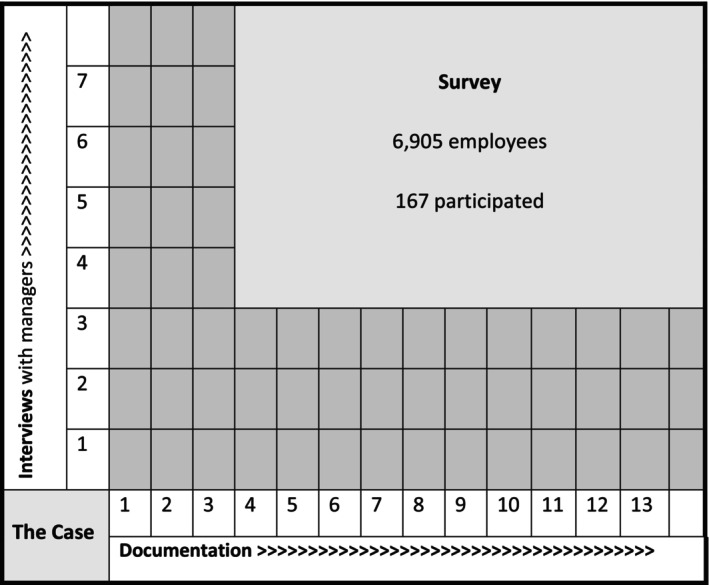
CSR—Methods used to examine the case study.

The project was funded by the University of Essex and received HRA ethical approval REC REF: 22/HRA/0590. Methods included an online survey of all employees, interviews with managers across the organisation and a review of workplace documentation including policies and procedures (see Figure 1).

## 5 SURVEY

The aim of the survey was to understand the employee base, prevalence and severity of perimenopausal and menopausal symptoms among employees, examination of workplace factors that influence symptoms and severity, and how the organisation might help employees who are impacted by perimenopausal and menopausal symptoms. In this paper, an overview of the survey is presented to provide some context to the case and the survey analysis and findings are presented in another paper showing the analysis of the survey (paper accepted for publication).

The online survey accessible on a range of mobile devices and computer was based on self‐report, with 75 questions taking approximately 20–25 min to complete. These included demographic and health‐related questions and a range of validated tools that are used widely to measure health and well‐being. There were a range of health and well‐being questions including sleep quality, levels of depression and a rating scale for menopause comparing severity of symptoms and a range of relationship to work‐related variables.

Questions around work in the organisation included role, working hours, work status, how long they have worked, shift patterns, flexibility, job satisfaction and level of job stress. There were questions around the work environment, work and job demands, control, support from manager and peers, relationships at work and role. Job performance included questions on presenteeism, absences related to perimenopause or menopause symptoms and leaving work early or arriving late for work. There were questions on turnover intentions: whether reducing hours, intending to leave workforce or the organisation itself.

Perimenopause and menopause questions related to the menstrual cycle, description, and duration of symptoms and seeking professional support. Other questions included who menopause symptoms were discussed with; whether receiving support or supporting someone else with menopause. The survey ended asking if employees were felt informed on the topic of menopause, whether they felt equipped to talk about menopause and asked what support would help.

## 6 INTERVIEWS

Seven managers were interviewed using a semi‐structured schedule conducted (JA) remotely by zoom minimising the risks associated with face‐to‐face visitation during the post‐pandemic phase. All interviews were transcribed verbatim ranging in duration from 10 to 26 min. The managers were employed across the organisation and length of service ranged from 3 to 32 years with an average of 15.1 years' service.

## 7 DOCUMENTATION

Thirteen documents were provided from the organisation (SS), published from 2017 to 2021 in a variety of formats and presented in different platforms transportable as electronic files across the organisation.

## 8 DATA ANALYSIS

Quantitative and qualitative data was generated and analysed in this case study. Descriptive and inferential analyses were performed (NA) on quantitative data to understand the sample and scales using the statistical software package SPSS of which the findings are presented elsewhere.

The qualitative data from the open‐ended survey responses and interviews (*n* = 7) were entered in NVivo (version 11) and thematically analysed (JA, CC) using the Braun and Clarke framework (2021). Content analysis (JA) of all documents was carried out with a narrative description completed. Reporting of this study was guided by the COREQ consolidated criteria for qualitative research (Tong et al., 2007).

## 9 FINDINGS

### 9.1 Survey

The demography of survey employees was 92% female, 8% male with the average employee aged 47.3 years (age range 23–66 years). Working status: 73% full‐time, 26% part‐time and 1% consisted of students and self‐employed and 61% (*n* = 102) of the participants had never smoked. The average height was 158.38 cm and average weight 76.77 kg, giving the average BMI of 30.6 (category obese).

While not all the survey data can be presented here significant findings showed a correlation between symptom severity, absenteeism and intention to leave the workplace. Of those reporting moderate or severe symptoms, at least 60% had taken time off, left early or been late for work in the preceding 4 weeks because of their symptoms. Of those with severe symptoms, 64% reported an intention to reduce working hours, 47% intended to leave the workforce and 44% intended to leave their employing organisation. The organisation reported that it has a majority female workforce of 81.9%, slightly higher than the UK average (Devine & Foley, 2020) with 40.6% aged between 41 and 55 years suggesting 33% working through perimenopause and menopause.

While the survey captured a small percentage of employees from this organisation it did however show significant findings. In the sample surveyed 66% reported experiencing menopausal symptoms with symptoms ranging from 38% reported mild symptoms, 49% moderate, and 13% reported experiencing severe symptoms. There was no correlation between shift pattern or number of hours worked and symptom severity, and 86% of employees reported that flexible working was available, although some employees and managers stated that some employees, especially those with patient‐facing roles, could not enjoy flexible working, while 49% reported being able to take a break when they felt it necessary.

Another finding reported was the association between symptom severity and anxiety and depression, with those experiencing severe symptoms most likely to score higher for anxiety and depression; a symptom severity increased with work‐related stress.

With lifestyle factors, more severe symptoms were reported among smokers, ex‐smokers and those who vape. Those who drank alcohol experienced more severe menopausal symptoms. To help manage symptoms, women reported using HRT, herbal remedies and contraception to help with menopausal symptoms. A correlation between exercise or BMI and symptom severity was not apparent, although employees reported using mindfulness, exercise and dietary changes to help reduce symptom severity. Some reported acceptance of menopause symptoms as a management strategy: *‘Acceptance of the changes I am going through reduces the anxiety’*.

### 9.2 Interviews

The data from the interviews were analysed and organised into two themes and six sub‐themes presented in Table 1 and discussed below.

**TABLE 1:** Themes and sub‐themes.

Themes	Access to support	Culture of menopause
	*‘more awareness and support’*	‘*you have just got to put up with it’*
Sub‐themes	Time poor	Taboo
	Support mechanisms	Labelling menopause
	Unsure of symptoms	Performance

### 9.3 Theme 1—Access to support ‘more awareness and support’

Employees reported feeling supported by both their colleagues and line managers in the workplace, with 47% reporting they could always approach their manager with work‐related issues. But only 6% of managers reported being asked by employees for support with menopause‐related issues.

M2 ‘I think that line manager relationship is really key, and I think if a person feels like are able to open a conversation about it to talk about the changes that they need then to do to try that’.

M4 ‘it really depends on the symptoms that they're having and what is adjustable in the workplace. You know I'm quite a laid back, not laid back, but easy going, I'm quite approachable, so you know I know the women, or you know any person that struggling with that would be able to come and tell me’.

M6 ‘I've seen this stuff coming out from the health and wellbeing team. And that's certainly something that I would direct them [staff] towards because it is the stuff that's right as far as I've been aware hasn't been directed towards managers has been directed towards staff themselves’.

M7 ‘We want to make sure that they're safe so whatever they're doing that they are safe and doing what they're doing. I think one would have to seek, because there's the emotional side is also physical side so occupation health, and whatever support is needed both emotional and any reasonable adjustments’.

Most employees were unsure of symptoms often referring to it as something else, others did not feel informed, nor did they feel equipped to support others experiencing menopause.

M1 ‘It is probably being called something else. It could be by being sort of masked you know people talk about migraines and headaches …’

M2 ‘I think that people are going to be taking, while they're going to either take days off sick or need to take days off sick and then not doing that, so they'll be working through their symptoms, maybe not being productive and where they want to, and also with absenteeism if they are going through the menopause and they haven't really explained that to the manager’.

M5 ‘I think they might fear that people would say, Oh, you know, everybody has menopause you've just got to put up with it so it's possible that they have taken or even that the person themselves hasn't recognized what this is’.

M7 ‘Menopause affects people in such a vast different range and some people don't even realize they're going through menopause, because it's just one of those things and there are other people that can affect them bad’.

Managers were largely pulling on personal knowledge and experiences of menopause, rather than any formal training, to help employees. Younger male and female managers reported that they would first seek help from colleagues who had experienced menopause to guide their support of others.

M1 ‘Nope. I say awareness, only what you pick up from various social media but not within work’.

M2 ‘the first thing I would do is look at I would personally look at what reasonable adjustments they might need because and became aware that menopause can now be seen as disability’.

M5 ‘I don't know I can only surmise that if you're suffering from perimenopause or menopause symptoms, if you, in general, you may be less able to cope with other things that you might otherwise have shrugged off. I would think there's probably a definite impact on mental health, but I don't know specifically’.

M7 ‘For me it's about allowing them to feel comfortable to come talk to me, but I think, I've tried to think, I'm like well, I've never had anybody come and talk to me about it, and I think sometimes it's a bit taboo for women, even to tell other women’.

Both employees and managers reported being unable to devote sufficient time to undertake menopause‐related training or to attend workplace menopause support sessions. Those that were able to attend support sessions reported that they helped with coping with menopause.

M1 ‘I have always promoted it and have been proactive with flexible working and trying to encourage that work life balance and support available’.

M2 ‘It comes back to those reasonable adjustments again if people can, I don't know, maybe have if they need a little bit more training, do something different, but still stay in work, then that needs to that needs to happen’.

M6 ‘There's definitely space for a lot more work around this topic just thinking of my own awareness, but just think of my own awareness of it and stuff that specifically targeted towards managers and also how to make that visible’.

Accessible formats of training and more workplace support mechanisms were requested by both employees and managers, with suggestions of online training modules and workshops with ‘lived experiences’ playing a role in delivery.

### 9.4 Theme 2—Culture of menopause ‘you've just got to put up with it’

Employees reported feeling uncomfortable discussing menopause with their managers. In comparison to the survey data, the interviews illustrated a different narrative where symptom severity could not be attributed to these factors and felt employees may not be honest about their true reasons causing absenteeism or poor performance as they often mask the real cause of symptoms.

M5 ‘I think perhaps, at least get people prepared to have those conversations. [There's] fear if you've not experienced symptoms and you're not likely to. It might be quite difficult to start having that conversation’.

M2 ‘You know running around on wards may not be the best idea for someone who is menopausal and has symptoms these are going to be a barrier, so can they do desk work, you know why we can't think creatively about this’.

A culture of menopause existed among both managers and employees, with managers feeling unable to broach the subject with employees who may be experiencing menopausal symptoms with menopause perceived a ‘taboo’ subject.

M2 ‘We know that we're a diverse trust and within some communities HRT still is a taboo subject. And if we're the people who are seen as you know, leaders in health, we're the people that ethnic minority people see daily, we should be having those conversations’.

M4 ‘I heard about menopause from my mum, and she had a really bad time … but other staff have come to me to discuss symptoms and how it affects them. We just see how it affects their work and work with the person’.

M5 ‘They might not tell me that's what the problem is because of stigma…I think they might fear that people would say, ‘Oh, you know, everybody has menopause you've just got to put up with it, so it's possible even that the person themselves hasn't recognised that this is a symptom ‐ they're just feeling terrible, and they don't know how’.

These themes show the nature of support from managers; but shows managers draw on own experiences. There was awareness of a new policy coming on board but otherwise no knowledge of other workplace polices, a mixed response on workplace support citing workshops and seminars as solutions; yet there was concern for the overall safety and welfare of employees who experienced symptoms of menopause but was discussed in terms of safety, disability and the ability to make adjustments for the employee if needed. Unfortunately, a culture of ‘*you've just got to put up with it’* exists within the organisation.

## 10 DOCUMENTATION

All documents and resources scrutinised as part of the study and tabulated for analysis to understand the content and extent of menopause information. The data covered one policy and documents that were available to support employed staff and managers in the organisation at the time.

This data included one draft workplace policy and 12 other documents and resources. Of these documents, there were flyers (*n* = 2) for past face‐to‐face menopause training events promoting menopause in lunch and learn sessions. There were copies of past presentations (*n* = 3) to employees and managers given by the same external consultant covering broadly the same topics on menopause physiology, the importance of diet and exercise, sexual health, contraception and availability of HRT. There were some other resources documents from an external human resource and research and development company offering generic workplace resources and recommendations, however not specific to the healthcare environment displayed a corporate feel, using images of women in business attire and largely grey and blue tones. One other resource advocated talking about menopause, and not making assumptions. It used a quote: ‘many women will continue to suffer in silence unless we break the taboo and start talking openly about the menopause at work’.

## 11 DISCUSSION

CSR explored the organisational culture of the workplace in terms of support and well‐being for staff experiencing perimenopausal and menopausal symptoms. Employees (66%) reported experiencing menopausal symptoms ranging from mild symptoms (38%), moderate (49%), and 13% reported experiencing severe symptoms impacting work and home life. These rates align with those reported nationally in Bazeley et al. (2022) study of menopause in the workplace.

Of those reporting moderate or severe symptoms, at least 60% had taken time off, left early or been late for work in the preceding 4 weeks, because of their symptoms. Of those with severe symptoms, 64% reported an intention to reduce working hours, 47% intended to leave the workforce, and 44% intended to leave their employing organisation. This organisation seems to be ambivalent of its environment and its effect on employees despite the fact rightly or wrongly assuming those working in a healthcare environment would be more prepared for perimenopause and menopause systems. As one manager put it *‘we have an ageing population and its impact in the workforce is only being recently seen as we have more people working later in life and we have not had to deal with this before*’.

Through the documentation the organisation has evidence of information sessions, seminars and policy development from various departments across the organisation; however, data from interviews and the employee survey showed variable levels of engagement, and furthermore reflected in employee lack of knowledge of menopausal symptoms. Managing perimenopause and postmenopausal health is an issue for all areas in health care and training for healthcare professionals should include menopausal health in their curriculum (Rees et al., 2021). There was certainly a mixed response from the survey and managers' views on and about symptoms of menopause. While some felt it was discussed openly in open forums or open places like break times, the managers generally felt they dealt with very few employees on a one‐to‐one basis, although a few examples of reasonable adjustments were discussed. Some managers and employees appeared to be aware of the most common menopausal symptoms such as hot flushes and lack of sleep but not around the many other symptoms such as anxiety, fatigue, joint pain, headaches, mood swings and brain fog (NICE, 2022).

Interestingly, it appears to be quite different from the individual perspective with few employees seeking support, and some managers thought other symptoms may mask menopausal symptoms and felt that employees rather that labelling as menopause would be fearful of what their organisation might say or do, and consequently influencing rates of absentiseem. This study also found that there appeared a misunderstanding around the term presenteeism and what it meant from both managers and employees. Presenteeism or employees working when they are ill or displaying symptoms is an increasing fact driven through a range of issues: obligation of role, stress or financial concerns (NHS England, 2022).

The interviews with managers shared scope on flexible working depending on employee role. While there was discussion around adjustments and approachability, it was felt that employees might find it difficult to approach managers, occupational health or GPs. Arguably menopause conversations are needed in the workplace to remove these barriers. Beck et al. (2020) survey of public sector roles found similar findings with some employees responding that they might be experiencing menopausal symptoms yet reflecting how they themselves lack knowledge of their own menopause status and contributing furthermore to the poor discourse of menopause. To further highlight this issue, Aljumah et al. (2023) online survey of 829 women to evaluate women's attitudes and knowledge of menopause found 94.1% of women have never been taught about menopause at school and 49.0% did not feel informed about menopause.

Survey data reported employees feeling uncomfortable discussing menopause with their managers, whereas the interviews illustrated a different narrative with managers perceiving themselves to be approachable. Beck et al. (2020) survey found that 77% would like information on menopause provided at work; suggested that environmental factors compound individual symptoms and how they managed at work. Interviewed managers felt they need more preparation to support employees and commented that staff might remain fearful or call their symptoms something else or may not know their symptoms. Beck et al. (2020) and Bazeley et al. (2022) work in the Fawcett report both corroborate these issues and the need for menopause awareness at work.

The interview data showed if symptoms were reported managers would discuss needs and would make necessary adjustments but felt very few employees came to speak about menopause. Newson (2021) workplace survey found that around 10% of menopausal women resign due to their menopausal symptoms, with 19% reducing their hours and 18% had taken more than 8 weeks off work due to their symptoms. Organisations need to monitor their workforce, specifically looking at age, if there are significant age profiles that may need attention and provide focused health and well‐being support in accessible formats to raise awareness. This case study has a slightly higher than the UK average female workforce with 40.6% aged between 41 and 55 years.

The culture of menopause needs to change and as it is not going away. Menopause needs to be treated differently, firstly, menopause as an issue for a growing older workforce working longer and secondly menopause is an issue that includes everyone needs to know about. This organisation employed a majority female workforce of 81.9%, higher than the national average with 78% in healthcare settings followed by 70% in education (Devine & Foley, 2020). A third of this organisation's workforce (40.6%) are aged between 41 and 55 years and working through perimenopause and menopause. As this rate is higher than other similar employers within the sector, the organisation needs to have clear protocol and policies in place for support, wider education and recognition of needs in the transition through perimenopause and menopause (Rees et al., 2021).

Healthcare environments are known to have significant stress and this study found correlation with high levels of stress and anxiety. Together with added pressures of the covid pandemic, those working in healthcare settings are predisposed to more severe menopausal symptoms. The interviews also saw that managers typically found employees want to continue to work despite their symptoms and therefore there may be some contradiction or just the need to recognise that those suffering menopause symptoms need support in the workplace. This includes learning and talking about their symptoms, learning to track symptoms and getting help or signposting to treat their symptoms (Cronin, 2021).

Several limitations included the low survey response rate, although this was reported to be in‐line with organisational norms and comparatively higher response rate than Hardy et al. (2018) national study. Employees in healthcare settings tend to be over‐surveyed possibly contributing to the low response rate. Additionally, the study was conducted post‐pandemic where healthcare organisations were facing unprecedented demands, after the Davina high‐profile TV documentary and during HRT shortages.

## 12 CONCLUSIONS

This study provided an in‐depth snapshot of a case, attempting to explore and understand the organisational culture of the workplace in terms of support and well‐being for staff experiencing perimenopausal and menopausal symptoms.

CSR provided an in‐depth analysis of this organisation showing a female majority workforce and a third working through perimenopause and menopause. This organisation has a majority female workforce with a third of the organisation working through perimenopause and menopause. Both employees and managers have disclosed and observed a range of issues, the organisation needs a joined‐up approach and the culture of menopause needs to change with good conversation where employees can talk, track and treat their symptoms. All staff need to understand menopause through education for themselves and others; and recognise that this type of organisation can be stressful for employees and may exacerbate symptoms of perimenopause and menopause.

## AUTHOR CONTRIBUTIONS

CC developed the concept, project design, contributed to analysis and drafted the manuscript. JA was a researcher employed for the duration of the project and conducted qualitative analysis. Statistical analysis was done by NA and contributed the review of the manuscript. The study site co‐ordinator on the project was SS.

## FUNDING INFORMATION

This project was funded by the University of Essex PVC‐R Fund.

## CONFLICT OF INTEREST STATEMENT

None declared.

## ETHICS STATEMENT

This study received HRA ethical approval [22/HRA/0590].

## Data Availability

Research data are not shared.
